# Preschool Wheezing: Trajectories and Long-Term Treatment

**DOI:** 10.3389/fped.2020.00240

**Published:** 2020-05-12

**Authors:** Valentina Fainardi, Angelica Santoro, Carlo Caffarelli

**Affiliations:** Clinica Pediatrica, Department of Medicine and Surgery, University of Parma, Parma, Italy

**Keywords:** preschool wheezing, phenotype, asthma, therapy, inhaled corticosteroids, skin prick test, allergy, infection

## Abstract

Wheezing is very common in infancy affecting one in three children during the first 3 years of life. Several wheeze phenotypes have been identified and most rely on temporal pattern of symptoms. Assessing the risk of asthma development is difficult. Factors predisposing to onset and persistence of wheezing such as breastfeeding, atopy, indoor allergen exposure, environmental tobacco smoke and viral infections are analyzed. Inhaled corticosteroids are recommended as first choice of controller treatment in all preschool children irrespective of phenotype, but they are particularly beneficial in terms of fewer exacerbations in atopic children. Other therapeutic options include the addition of montelukast or the intermittent use of inhaled corticosteroids. Overuse of inhaled steroids must be avoided. Therefore, adherence to treatment and correct administration of the medications need to be checked at every visit.

## Introduction

In preschool children, wheezing is very heterogeneous and evidence on pathophysiology and treatment is scant (1). Preschool wheezing should be considered an umbrella term for distinctive diseases with different observable and measurable features (phenotypes). Each phenotype may be the result of different endotypes, described by a mechanism that links clinical features with a molecular pathway. Different approaches have been used in the search of wheezing phenotypes. The selection of observable variables can be driven by the investigator or by data. The design can be cross-sectional or longitudinal. In this review, we discuss knowledge on expression of preschool wheezing phenotypes and their value regarding outcomes, prognosis and long-term treatment.

## Wheeze Trajectories

About one in three children experiences wheezing during the first 3 years of life (2, 3) but not all will develop asthma in later age (4). Several phenotypes of preschool wheeze that mostly rely on temporal pattern of symptoms have been proposed to identify individuals at risk for asthma onset at school age (1). Other variables including symptoms, bronchial inflammation, allergic sensitization, medication use, and lung function have been also used for classifying subgroups in relation to timing of occurrence and resolution of wheezing.

Birth longitudinal cohort studies have reported numbers of preschool wheeze phenotypes varying from 2 to 6. Three phenotypes are more common (3): (a) *transient early wheeze* occurs before the age of 3 years and resolves by age of 6 years without lung function impairment; (b) *late-onset wheeze* develops after 3 years of age and persists in childhood, it is linked to atopy, and, in some studies, it is associated to reduced lung function and high bronchial hyperresponsiveness;

(c) *persistent wheeze* starts in early life before 3 years of age and it is associated with atopy, high IgE levels, early allergen sensitization and diminished lung function by school age (3, 5–13). In Project Viva, a prebirth cohort study in Massachusetts (14), transient-early wheeze and persistent wheeze were found. Children with persistent wheezing or late-onset wheezing more frequently have asthma in adolescence (15).

In the Avon Longitudinal Study of Parents and Children (ALSPAC) birth cohort, two more phenotypes were identified at 9 years of age (9, 16): (a) *prolonged early wheeze* group (onset between 6 and 54 months of age), not associated with airborne allergen sensitization and weakly associated with higher airway responsiveness and impaired lung function; (b) *intermediate-onset wheeze* group (onset between 18 and 42 months) with persisting symptoms, atopy, poor lung function and at more risk of developing asthma in childhood. At 15 years of age, symptoms continued in school age-onset persisting phenotype and in late childhood onset persisting phenotype and in continuous wheeze group and they were associated with bronchodilator reversibility, fractioned exhaled nitric oxide (FeNO) levels ≥35 ppb and impaired pulmonary function (16, 17). Finally, preschool wheeze persisting after 18 months of age is a risk factor for asthma, reduced pulmonary function and higher FeNO value in adolescence (16). Wheezing phenotypes identified in the ALSPAC study have been confirmed by the PIAMA study with the exception of the *prolonged early wheeze* phenotype (13).

In the Manchester Asthma and Allergy Study (MAAS) a new phenotype named *persistent troublesome wheeze*, has been characterized. Children in this category had more frequent exacerbations, hospitalizations, and unscheduled visits, reduced lung function, higher bronchial hyperreactive airways, higher IgE levels to inhalant allergens in comparison with other phenotypes. A sum of medium wheal diameter of skin prick tests (SPT) ≥ 10 mm and a history of exacerbations by age 3 years predicted persistent troublesome wheeze (8). In the MAAS cohort (18), new phenotypes of children with severe wheeze exacerbations were found when trajectories were examined at 8 years of age and at adolescence. Early-onset frequent exacerbations compared to infrequent exacerbations or no exacerbation had shorter breastfeeding, lower lung function, higher FeNO.

Despite the limitations in categorizing preschool wheeze (1), wheeze trajectories have been used to determine several models for predicting the onset of asthma at school age in high-risk population or in general population (4, 19–31). Unfortunately, the clinical value of predictive indexes for ruling out the development of asthma at school age has been shown to be too low to be adopted in clinical practice (32–34). However, original API is a very promising predictive tool, since it has an appreciable likelihood ratio (~7.4) and it consists only of four clinical parameters and blood eosinophil count (34). Over the last years, many groups attempted to update these definitions and identify more detailed phenotypes using for example latent class analysis (LCA) technique (17, 35, 36) but results from a clinical point of view were rather disappointing.

Several factors that can be potentially modified by simple interventions may change wheeze trajectories. In the Canadian Asthma Primary Prevention Study, pre-school wheezers were classified as *early-transient* (10%) who did not develop asthma, *low-progressive* (69%) who gradually developed asthma over time, and *early-persistent* (21%) with higher risk of asthma occurrence (37). Only in the early-persistent group wheezing prevalence was significantly reduced by intervention including breastfeeding, delayed weaning, avoidance of house dust mite, pets and environmental tobacco smoke.

In the Urban Environment and Childhood Asthma birth cohort, five phenotypes were described (38). Asthma developed in the 60–70% of children with high wheeze/low atopy and high wheeze/high atopy while it was infrequent in low wheeze/high atopy (14%) and in low wheeze/low atopy (1%) and absent in transient wheeze/low atopy. Environmental exposures in early life differed among phenotypes. Maternal stress and depression, prenatal smoke exposure were associated with the high wheeze/low atopy group. Indoor allergen exposure was high in the low wheeze/low atopy group and low in the two high wheeze phenotypes. Household microbial richness and diversity were low in high wheeze/high atopy and highest in transient wheeze/low atopy.

In the French Longitudinal Study of Children, wheezing infants at 2 months of age with 1 or 2 siblings, nocturnal cough, respiratory distress, extreme bronchial secretion, reflux, maternal asthma and maternal smoking during pregnancy were found to be at risk for persistent wheezing at 1 year of age (39).

## Virus and Allergy

In the past, an ERS task force (40) proposed a simple clinical classification of wheezing as “*episodic viral wheezing*” (EVW) or “*multiple trigger wheezing*” (MTW) based on triggers and symptoms. A wheezing typically exacerbated by a viral upper respiratory tract infection with few or no symptoms in the interval between the episodes was described as EVW, the commonest phenotype between 1 and 5 years of age. Children who have symptoms that resemble asthma with wheezing also between respiratory infections and during activity, crying or laughing show the phenotype traditionally called MTW. Children with MTW are usually atopic and may have a family history for asthma. The usefulness of such classification of wheezing in clinical practice is hampered by several factors. It does not consider that is based on patient characteristics at the time of examination (symptom pattern, trigger factors, allergic features…) that change over time (41). Another limitation is that does not take into account the severity of the episodes and cannot identify children responding to specific treatments (42). Furthermore, the two phenotypes can have similarities and cover different endotypes.

In young children, the endotype is difficult to assess because airway samples like bronchoalveolar lavage and bronchial biopsies are not easy to obtain (43). A research performed on a small sample of preschool children with severe wheezing suggested that EVW might be associated to airway neutrophilia and to airway microbiota dominated by Moraxella although the statistical significance was not reached (44). To date, in young children no study has linked lower airway inflammation or infection to a particular wheeze phenotype (1).

Regarding the Th2 high endotype, Fitzpatrick AM et al. (43) distinguished by LCA four latent classes of recurrent preschool wheeze on the basis of type-2 inflammatory features including blood eosinophils, atopic eczema, aeroallergen and food sensitization and/or pet exposures. The probability of exacerbation was greater in children with sensitization and indoor pet exposure (latent class 2) and children with multiple sensitization and eczema (latent class 4). Overall in children sensitized to aeroallergens this risk was higher than in children with minimal sensitization (latent class 1- sporadic eczema, low blood eosinophils, low serum IgE levels, >90% with no sensitization to aeroallergen and food) or with sensitization plus tobacco smoke exposure (latent class 3) with no exposure to pets. It is not surprising that children with atopic eczema are at risk for exacerbation because airway inflammation has been found in children with atopic eczema without asthma symptoms (45).

Sensitization to foods is more frequent than airborne allergy under 5 years of age (46). It has been shown that asthma that remains over time and severe asthma attacks are more common in children with food allergy and depends on number of offending foods (47). These findings suggest that it may be worthwhile to perform skin prick tests to foods in patients with wheezing and/or atopic eczema (48).

Since atopy is a risk factor for asthma, it has been proposed that probiotics may potentially stimulate Th1 and decrease allergic asthma. Accordingly, there are some data that probiotics supplementation may be of benefit in the prevention of wheeze or asthma (49). Another potential approach is to investigate strategies that prevent viral respiratory tract infections to reduce the development of asthma. However, the impact of probiotics or immunostimulant agents on recurrence of such infections seems to be small (50, 51).

## Long-Term Therapeutic Approach and Phenotypes

To date, the optimal therapy for preventing recurrent episodes of wheezing in preschool children is not well-defined and remains a matter of study and debate (52–54). In preschool children, inhaled corticosteroids (ICS) are recommended as first choice of controller treatment in all subjects with frequent exacerbations irrespective of phenotype or endotype (55, 56).

A systematic review of 29 randomized controlled trials (RCTs) in preschool children (no 3,592) showed that ICS reduced the frequency of wheeze episodes independently from phenotype and atopy (57). Similarly, a more recent meta-analysis including 15 studies for a total number of 3,278 children aged ≤6 years demonstrated that daily medium dose ICS reduced the risk of exacerbations by 30% compared to placebo. Moreover, when the analysis was performed in the subgroup of children with persistent asthma the risk of exacerbation was reduced by 44% (53).

The question arises on which preschool children with wheeze may benefit from ICS. The finding of peripheral blood eosinophilia, that in this age group reflects higher airway eosinophilia (58), can suggest the possible response to a continuous therapy with ICS (57). Accordingly, the Individualized Therapy for Asthma in Toddlers (INFANT) trial showed that daily therapy with ICS is beneficial in terms of fewer exacerbations and better symptom control in children aged 12–59 months with aeroallergen sensitization and high blood eosinophils (≥300/μL) (54). Along this line, daily ICS were found to significantly reduce future exacerbations in children with sensitization and indoor pet exposure (latent class 2) and children with multiple sensitization and eczema (latent class 4), but not in children with minimal sensitization (latent class 1) or children with sensitization and second hand tobacco smoke exposure (latent class 3) (43). Therefore, children with MTW experiencing symptoms most days of the week and children with frequent episodes of wheezing (every 6–8 weeks) and/or severe exacerbations requiring systemic corticosteroids irrespective of wheezing phenotype are good candidates to start a trial (at least 6–8 weeks) with daily very low or low dose ICS (Table 1) (55, 57, 59, 60). Other guidelines suggest beginning with moderate dose ICS (56). After a therapeutic trial with ICS for 8–12 weeks, the child should be reassessed to see if symptoms have resolved and treatment can be tapered down or interrupted. Subsequently, ICS treatment should be given again when needed to control symptoms.

**Table 1:** Inhaled corticosteroid by recommended dose.

**Inhaled corticosteroid**	**Very low dose**	**Low dose**	**Medium dose**
Fluticasone propionate HFA	50 mcg one puff twice a day	50 mcg two puffs twice a day	125 mcg two puffs twice a day
Beclomethasone dipropionate HFA	50 mcg two puffs twice a day	100 mcg two puffs twice a day	200 mcg two puffs twice a day
Budesonide nebulized	250 mcg/day	500 mcg/day	>500–1,000 mcg/day

*Fluticasone and beclomethasone are considered as pressurized metered dose inhalers (pMDI) with spacer. Adapted from BTS guidelines and GINA recommendations (55, 59). HFA, Hydrofluoroalkane propellant*.

In children with mild symptoms aged 6–11 years, the updated GINA recommendations (59) showed a change in the management of wheezing recommending the use of ICS with short acting beta-agonists (SABA) as needed or regular ICS with as needed SABA. However, trials on this issue are lacking in preschool children. The INFANT study showed that some children benefited from as-needed ICS/albuterol as daily ICS or daily montelukast plus as-needed albuterol. Further studies are warranted to clarify endotypes that are associated with response to treatment. If the initial treatment with low dose ICS is not enough to reach good control, various alternatives are available (59). The preferred option is doubling the dose (see “medium dose” in Table 1). The beneficial effects of daily ICS have been shown up to 400 mcg/day of beclomethasone equivalent (or 200 mcg/day fluticasone) via pressurized metered-dose inhaler with spacer without any further improvement for higher doses (61).

Before increasing or changing the therapy, checking that the medication is regularly administered and that inhalers are correctly used is mandatory. Adherence to ICS is crucial in achieving and maintaining control of symptoms in young children with wheeze. In an observational study in asthmatic children aged 2–6 years adherence to ICS was measured daily for 12 months showing that adherence was the only determinant significantly associated with asthma control (62). Moreover, if the child is not too sick to use inhalers, an MDI/spacer combination is the gold standard strategy to deliver corticosteroids and bronchodilators and inhalation technique should regularly checked (63). In those children where regular intake of low dose ICS plus as-needed SABA fails to control symptoms or exacerbations, the preferred option is increasing the dose of ICS (56, 59), after having excluded other diagnosis, allergen exposure and check adherence to treatment. Latest recommendations suggest that in children with EVW and no signs of atopy adding a leukotriene receptor antagonist (LTRA), rather than doubling the dose of ICS may be an option (59, 60). However, RCTs comparing the association of LTRA plus ICS vs. ICS alone need to be performed before physicians can recommend this combination in preschoolers. Moreover, montelukast may cause several adverse effects and the US Food and Drug Administration has required to add a boxed warning about mental health side effects. So, the risk-benefit balance of montelukast may not be favorable in some patients, particularly when the symptoms may be mild and controlled with other drugs (64). When medium dose ICS does not control symptoms other add-on therapies [LTRA, long acting beta-agonists (LABA)] in children >4 years of age) may be considered. Further options include the addition of theophylline or oral corticosteroids (OCS). Allergen-specific immunotherapy can be a therapeutic opportunity (65, 66). It is recommended that all children, including asthmatics, timely perform vaccinations for infectious diseases and this is feasible even if they have had allergic reactions to vaccines (67). It is important that children suffering from asthma receive influenza and pneumococcal vaccinations. The risk for invasive pneumococcal disease is declined in asthmatics after the introduction of pneumococcal vaccination. However, Castro-Rodriguez JA et al. (68) documented that even among children who received pneumococcal conjugate vaccine, asthmatic children were at higher risk for invasive pneumococcal disease and pneumonia in comparison with non-asthmatic children (68). Therefore, trials are warranted to assess whether asthmatics >2 years of age should receive a supplemental 23-valent pneumococcal polysaccharide vaccination after pneumococcal conjugate vaccine regardless the intake of high-dose OCS as reported in the current guidelines in US. Recently the US Food and Drug Administration and the European Medicines Agency extended the use of tiotropium as add-on maintenance treatment in children >6 years of age. Vrijlandt et al. (69) has found that tiotropium add-on therapy reduced exacerbations in preschool asthma symptoms not controlled by ICS and LABA or montelukast. Unfortunately, in this study an association of efficacy of tiotropium with Th2 high endotype was not determined.

## Other Baseline Treatments

The use of intermittent high dose of ICS is an alternative option in preschool wheeze. In preschool children with EVW who are asymptomatic between the episodes the intermittent use of high doses of steroids (for example beclomethasone dipropionate 400 mcg twice daily, fluticasone propionate 250 mcg twice daily or budesonide 1 mg twice daily) at the first sign of respiratory tract infection and continued for up to 7–10 days may be the preferred choice (53). Interestingly, this approach is not inferior to daily steroids also in children at risk of developing asthma (70). A Cochrane review comparing high ICS doses demonstrated a reduction in requirement for OCS compared to placebo (71). Ducharme et al. (52) in a meta-analysis of 4 RCTs (no. 1,024) reported that compared to placebo, high doses of ICS reduced the risk of exacerbations requiring OCS in preschoolers with moderate or severe episodic viral wheeze. A further Cochrane meta-analysis comparing intermittent ICS with placebo in 490 preschool children with persistent asthma found a reduction in the need of rescue OCS of almost 50% (72). A more recent meta-analysis of RCTs showed that, compared to placebo, the use of intermittent ICS for at least 7–10 days in children with episodic viral wheeze reduced the risk of exacerbation by 36%. Interestingly, no significant differences in severe exacerbation rate were found when this strategy was compared to daily ICS (53). However, the comparison between intermittent and continuous therapy yielded controversial results. In the study by Papi et al. (73) intermittent therapy with nebulized beclomethasone 800 mcg/day was not inferior to daily low dose ICS in symptom control but exacerbation rate was lower with continuous therapy. In contrast, the study by Zeiger et al. (70) showed no difference between the two treatments in exacerbation frequency but a significant lower exposure to steroids with intermittent modality. A 2013 Cochrane meta-analysis including two trials in preschool children with persistent asthma found inconclusive evidence about the efficacy of intermittent vs. daily ICS (74). Though, the meta-analysis by Castro-Rodriguez evaluating daily and intermittent ICS in preschoolers, school-aged children and adults with persistent wheezing and mild to moderate persistent asthma showed no statistically significant difference in the rate of asthma exacerbations but more free asthma days in those on daily preventing therapy (75).

When the use of inhaled steroids is intermittent the prescribed dose should be enough high to be successful because low-moderate doses seem to be ineffective (76, 77). Indeed, in an Italian RCT the intermittent use of 400 mcg of nebulized beclomethasone twice a day for 10 days did not affect neither the evolution nor the symptoms of the exacerbation in preschool children with viral wheeze (78). However, high doses of steroids imply the use of the highest licensed dose and this is particularly important to avoid an overload of these medications since in this age range children can experience a significant high number of viral exacerbations along the year (76).

Another option for the baseline treatment can be the regular therapy with montelukast, a leukotriene antagonist that modestly reduces symptoms and need for rescue bronchodilator or OCS compared with placebo (79). However, the strength of evidence to support the use of montelukast is limited by the difficulty of the studies in distinguishing EVW from an asthma-like phenotype like MTW. A systematic review and meta-analysis including two RCTs on the use of intermittent or continuous montelukast in children with episodic viral wheeze and no interval symptoms showed that there is no statistically significant difference between the treatment with montelukast and placebo in the number of exacerbations requiring OCS (80). Krawiec et al. (81) reported a similar result in 70 children aged 36 months to 6 years where daily therapy with montelukast did not have any influence in preventing wheeze episodes over 1 year. Furthermore, in comparative studies montelukast is less efficacious than daily steroids in reducing wheeze exacerbations requiring OCS (82). The use of intermittent montelukast in preschool children with wheeze is likely effective only in a specific genotype-dependent subgroup carrying the promoter 5/5 ALOX5 (83).

## Limitation OF ICS

The effectiveness of ICS for preventing attacks of wheezing in preschool children is always questioned. Very recently, a UK cohort study of children aged 1–5 years reported that initiation of the controller therapy with ICS did not result in fewer exacerbations compared to bronchodilators over 1 year. That said, the electronic data of this study might have represented a very general population of children with mild forms of wheezing since children on ICS before the age of 1 and those prescribed ICS or montelukast during the baseline year were excluded (84). On the other hand, a cohort study including children under 5 years recorded in the same country an increase in ICS prescriptions and treatment of exacerbations over the last 10 years (85). In interpreting these findings it should be considered that prescription of ICS is frequent in children with low tract respiratory infections who do not wheeze. Therefore, choosing the appropriate pharmacological treatment in children with airways disorders is still challenging, a correct diagnostic work up is crucial to avoid inadequate prescriptions (86, 87).

The treatment with continuous or intermittent ICS and the treatment with montelukast do not prevent the progression of any phenotype of preschool wheeze to asthma in later ages but aim to improve symptom control and reduce exacerbations (88–90). The main side effect of a long-term therapy with steroids is growth suppression (91), a topic that should be always discussed with parents. The effects of ICS on growth velocity are seen in pre-pubertal children in the first 1–2 years of treatment resulting in a decrease of 0.5–1 cm in height growth (88). This is not progressive or cumulative and one study on long-term outcomes of continuous ICS showed a difference of 0.7% in adult height (92). It is likely that intermittent treatment has less effect on linear growth because of the lower cumulative dose (53, 70, 74). However, asthma itself as the other chronic diseases, has transient negative impact on growth in children, but the final height is not affected. So, there is a need of trials that investigate whether adult height is reduced by ICS in asthmatics (93). The long-term use of ICS at high doses may have effects on hypothalamic-pituitary-adrenal axis, resulting in reduced adrenal cortisol production (94). However, many studies show a wide variability on the partial suppression of adrenal cortisol, depending on the individual susceptibility, the severity of asthma, the type of inhaled corticosteroid and the inhalation device used. In most cases the suppression is asymptomatic and life-threatening condition are extremely rare. Severe hypoglycaemia due to adrenal insufficiency is reported only in two cases of preschool children treated with inhaled fluticasone propionate at a dosage of 780 μg/m2/day (95).

In the first year of life, symptoms are triggered by viral or bacterial infections. Both atopic and non-atopic wheezers have no eosinophilic airway inflammation (96). Treatment options for controlling symptoms include trials with montelukast or beta2-agonists. Beta2-agonists should be stopped after 4–8 weeks and, when effective, they can be given if necessary. Regarding montelukast, a course of 2–3 months, should be regarded as useful for assessing the efficacy.

## Conclusions

Studies on preschool wheezing phenotypes are of little clinical help for identifying children who are more likely to develop asthma at school age. Drawbacks of such studies include heterogeneous results, with only partial consistency, overlapping features and instability of phenotypes over time. Disagreements between the results may be explained by different study populations, variations in approaches for selecting observational features, lack of uniformity in choosing type and number of variables. Regarding long-term treatment, ICS are the first-line therapy in preschool children with asthma or recurrent wheezing. With the limitations related to a classification based on temporal phenotype, daily ICS are the preferred choice in children with MTW and frequent symptoms, especially those with blood eosinophilia and aeroallergen sensitivity, while intermittent high dose ICS can be considered in children with EVW and no symptoms between the episodes. To date, there is no evidence to recommend the use of intermittent or daily montelukast.

## Author Contributions

VF, AS, and CC co-wrote the manuscript and approved the final version.

## Conflict of Interest

The authors declare that the research was conducted in the absence of any commercial or financial relationships that could be construed as a potential conflict of interest.

## Abbreviations

FeNO: fractioned exhaled nitric oxide
LCA: latent class analysis
EVW: episodic viral wheezing
MTW: multiple trigger wheezing
ICS: inhaled corticosteroids
RCTs: randomized controlled trials
LTRA: leukotriene receptor antagonist
LABA: long acting beta-agonists
SABA: short acting beta-agonists
OCS: oral corticosteroids.

## References

[1] Brand PL Caudri D Eber E Gaillard EA Garcia-Marcos L Hedlin G. Classification and pharmacological treatment of preschool wheezing: changes since 2008. Eur Respir J. (2014) 43:1172–7. 10.1183/09031936.00199913 24525447

[2] Bisgaard H Szefler S. Prevalence of asthma-like symptoms in young children. Pediatr Pulmonol. (2007) 42:723–8. 10.1002/ppul.20644 17598172

[3] Martinez FD Wright AL Taussig LM Holberg CJ Halonen M Morgan WJ. Asthma and wheezing in the first six years of life. N Engl J Med. (1995) 332:133–8. 10.1056/NEJM199501193320301 7800004

[4] Pescatore AM Dogaru CM Duembgen L Silverman M Gaillard EA Spycher BD A simple asthma prediction tool for preschool children with wheeze or cough. J Allergy Clin Immunol. (2014) 133: 111–8. 10.1016/j.jaci.2013.06.002 23891353

[5] Howard R Rattray M Prosperi M Custovic A. Distinguishing asthma phenotypes using machine learning approaches. Curr Allergy Asthma Rep. (2015) 15:38. 10.1007/s11882-015-0542-0 26143394 PMC4586004

[6] Belgrave DC Custovic A Simpson A. Characterizing wheeze phenotypes to identify endotypes of childhood asthma, and the implications for future management. Expert Rev Clin Immunol. (2013) 9:921–36. 10.1586/1744666X.2013.836450 24128156

[7] Deliu M Sperrin M Belgrave D Custovic A. Identification of asthma subtypes using clustering methodologies. Pulm Ther. (2016) 2:19–41. 10.1007/s41030-016-0017-z 27512723 PMC4959136

[8] Belgrave DCM Simpson A Semic-Jusufagic A Murray CS Buchan I Pickles A. Joint modeling of parentally reported and physician-confirmed wheeze identifies children with persistent troublesome wheezing. J Allergy Clin Immunol. (2013) 132:575–83.e12. 10.1016/j.jaci.2013.05.041 23906378

[9] Henderson J Granell R Heron J Sherriff A Simpson A Woodcock A. Associations of wheezing phenotypes in the first 6 years of life with atopy, lung function and airway responsiveness in mid-childhood. Thorax. (2008) 63:974–80. 10.1136/thx.2007.093187 18678704 PMC2582336

[10] Granell R Sterne JA Henderson J. Associations of different phenotypes of wheezing illness in early childhood with environmental variables implicated in the aetiology of asthma. PLoS ONE. (2012) 7:48359. 10.1371/journal.pone.0048359 23118993 PMC3485223

[11] Collins SA Pike KC Inskip HM Godfrey KM Roberts G Holloway JW. Validation of novel wheeze phenotypes using longitudinal airway function and atopic sensitization data in the first 6 years of life: evidence from the Southampton Women's survey. Pediatr Pulmonol. (2013) 48:683–92. 10.1002/ppul.22766 23401430 PMC3689612

[12] Rusconi F Galassi C Corbo GM Forastiere F Biggeri A Ciccone G. Risk factors for early, persistent, and late-onset wheezing in young children. Am J Respir Crit Care Med. (1999) 160:1617–22. 10.1164/ajrccm.160.5.9811002 10556130

[13] Savenije OE Granell R Caudri D Koppelman GH Smit HA Wijga A. Comparison of childhood wheezing phenotypes in 2 birth cohorts: ALSPAC and PIAMA. J Allergy Clin Immunol. (2011) 127:1505–12.e14. 10.1016/j.jaci.2011.02.002 21411131

[14] Sordillo JE Coull BA Rifas-Shiman SL Wu AC Lutz SM Hivert MF. Characterization of longitudinal wheeze phenotypes from infancy to adolescence in Project Viva, a prebirth cohort study. J Allergy Clin Immunol. (2020) 145:716–9. 10.1016/j.jaci.2019.10.026 31705908 PMC7010553

[15] Taussig LM Wright AL Holberg CJ Halonen M Morgan WJ Martinez FD. Tucson children's respiratory study: 1980 to present. J Allergy Clin Immunol. (2003) 111:661–75. 10.1067/mai.2003.162 12704342

[16] Duijts L Granell R Sterne JA Henderson AJ. Childhood wheezing phenotypes influence asthma, lung function and exhaled nitric oxide fraction in adolescence. Eur Respir J. (2016) 47:510–9. 10.1183/13993003.00718-2015 26647439

[17] Granell R Henderson J Sterne JA. Associations of wheezing phenotypes with late asthma outcomes in the avon longitudinal study of parents and children: a population-based birth cohort. J Allergy Clin Immunol. (2016) 138:1060–70. 10.1016/j.jaci.2016.01.046 27106203 PMC5052126

[18] Deliu M Fontanella S Haider S Sperrin M Geifman N Murray C. Longitudinal trajectories of severe wheeze exacerbations from infancy to school age and their association with early-life risk factors and late asthma outcomes. Clin Exp Allergy. (2020) 50:315–24. 10.1111/cea.13553 31876035 PMC7065181

[19] Castro-Rodríguez JA Holberg CJ Wright AL Martinez FD. A clinical index to define risk of asthma in young children with recurrent wheezing. Am J Respir Crit Care Med. (2000) 162:1403–6. 10.1164/ajrccm.162.4.9912111 11029352

[20] Castro-Rodriguez JA. The asthma predictive index: early diagnosis of asthma. Curr Opin Allergy Clin Immunol. (2011) 11:157–61. 10.1097/ACI.0b013e3283464c4a 21464709

[21] Guilbert TW Morgan WJ Krawiec M Lemanske RF Jr Sorkness C Szefler SJ. The Prevention of early asthma in kids study: design, rationale and methods for the childhood asthma research and education network. Control Clin Trials. (2004) 25:286–310. 10.1016/j.cct.2004.03.002 15157730

[22] Kurukulaaratchy RJ Matthews S Holgate ST Arshad SH. Predicting persistent disease among children who wheeze during early life. Eur Respir J. (2003) 22:767–71. 10.1183/09031936.03.00005903 14621083

[23] Devulapalli CS Carlsen KC Haland G Munthe-Kaas MC Pettersen M Mowinckel P. Severity of obstructive airways disease by age 2 years predicts asthma at 10 years of age. Thorax. (2008) 63:8–13. 10.1136/thx.2006.060616 17615086

[24] Lodrup Carlsen KC Soderstrom L Mowinckel P Haland G Pettersen M Munthe Kaas MC. Asthma prediction in school children; the value of combined IgE antibodies and obstructive airways disease severity score. Allergy. (2010) 65:1134–40. 10.1111/j.1398-9995.2010.02344.x 20219060

[25] Caudri D Wijga A Schipper CM Hoekstra M Postma DS Koppelman GH. Predicting the long-term prognosis of children with symptoms suggestive of asthma at preschool age. J Allergy Clin Immunol. (2009) 124:903–10.e1–7. 10.1016/j.jaci.2009.06.045 19665765

[26] Vial Dupuy A Amat F Pereira B Labbe A Just J. A simple tool to identify infants at high risk of mild to severe childhood asthma: the persistent asthma predictive score. J Asthma. (2011) 48:1015–21. 10.3109/02770903.2011.626481 22022892

[27] Matricardi PM Illi S Gruber C Keil T Nickel R Wahn U. Wheezing in childhood: incidence, longitudinal patterns and factors predicting persistence. Eur Respir J. (2008) 32:585–92. 10.1183/09031936.00066307 18480107

[28] Eysink PE ter Riet G Aalberse RC van Aalderen WM Roos CM van der Zee JS. Accuracy of specific IgE in the prediction of asthma: development of a scoring formula for general practice. Br J Gen Pract. (2005) 55:125–31. 15720934 PMC1463187

[29] Leonardi NA Spycher BD Strippoli MP Frey U Silverman M Kuehni CE. Validation of the Asthma Predictive Index and comparison with simpler clinical prediction rules. J Allergy Clin Immunol. (2011) 127:1466–72. 10.1016/j.jaci.2011.03.001 21453960

[30] Chang TS Lemanske RF Guilbert TW Gern JE Coen MH Evans MD. Evaluation of the modified asthma predictive index in high-risk preschool children. J Allergy Clin Immunol In Practice. (2013) 1:152–6. 10.1016/j.jaip.2012.10.008 24187656 PMC3811153

[31] Amin P Levin L Epstein T Ryan P LeMasters G Khurana-Hershey G Optimum predictors of childhood asthma: persistent wheeze or the asthma predictive index? J Allergy Clin Immunol Pract. (2014) 2:709–15. 10.1016/j.jaip.2014.08.009 25439361 PMC4254628

[32] Smit HA Pinart M Antó JM Keil T Bousquet J Carlsen KH. Childhood asthma prediction models: a systematic review. Lancet Respir Med. (2015) 3:973–84. 10.1016/S2213-2600(15)00428-2 26597131

[33] Savenije OE Kerkhof M Koppelman GH Postma DS. Predicting who will have asthma at school age among preschool children. J Allergy Clin Immunol. (2012) 130:325–31. 10.1016/j.jaci.2012.05.007 22704537

[34] Castro-Rodriguez JA Cifuentes L Martinez FD. Predicting asthma using clinical indexes. Front Pediatr. (2019) 7:320. 10.3389/fped.2019.00320 31463300 PMC6707805

[35] Hose AJ Depner M Illi S Lau S Keil T Wahn U. Latent class analysis reveals clinically relevant atopy and phenotypes in 2 birth cohorts. J Allergy Clin Immunol. (2017) 139:1935–45. 10.1016/j.jaci.2016.08.046 27771325

[36] Oksel C Granell R Mahmoud O Custovic A Henderson AJ. Causes of variability in latent phenotypes of childhood wheeze. J Allergy Clin Immunol. (2019) 143:1783–90. 10.1016/j.jaci.2018.10.059 30528616 PMC6505513

[37] Owora AH Becker AB Chan-Yeung M Chan ES Chooniedass R Ramsey C. Wheeze trajectories are modifiable through early-life intervention and predict asthma in adolescence. Pediatr Allergy Immunol. (2018) 29:612–21. 10.1111/pai.12922 29729041

[38] Bacharier LB Beigelman A Calatroni A Jackson DJ Gergen PJ O'Connor GT. Longitudinal phenotypes of respiratory health in a high-risk urban birth cohort. Am J Respir Crit Care Med. (2019) 199:71–82. 10.1164/rccm.201801-0190OC 30079758 PMC6353010

[39] Hallit S Leynaert B Delmas MC Rocchi S De Blic J Marguet C Wheezing phenotypes and risk factors in early life: the ELFE cohort. PLoS ONE. (2018) 13:e0201863 10.1371/journal.pone.0201863 29702689 PMC5922557

[40] Brand PL Baraldi E Bisgaard H Boner AL Castro-Rodriguez JA Custovic A. Definition, assessment and treatment of wheezing disorders in preschool children: an evidence-based approach. Eur Respir J. (2008) 32:1096–110. 10.1183/09031936.00002108 18827155

[41] van Wonderen KE Geskus RB van Aalderen WM Mohrs J Bindels PJ van der Mark LB. Stability and predictiveness of multiple trigger and episodic viral wheeze in preschoolers. Clin Exp Allergy. (2016) 46:837–47 10.1111/cea.12660 26464237

[42] Schultz A Brand PL. Episodic viral wheeze and multiple trigger wheeze in preschool children: a useful distinction for clinicians? Paediatr Respir Rev. (2011) 12:160–4. 10.1016/j.prrv.2011.01.008 21722843

[43] Fitzpatrick AM Bacharier LB Guilbert TW Jackson DJ Szefler SJ Beigelman A. Phenotypes of recurrent wheezing in preschool children: identification by latent class analysis and utility in prediction of future exacerbation. J Allergy Clin Immunol Pract. (2019) 7:915–24. 10.1016/j.jaip.2018.09.016 30267890 PMC6401237

[44] Robinson PFM Pattaroni C Cook J Gregory L Alonso AM Fleming LJ. Lower airway microbiota associates with inflammatory phenotype in severe preschool wheeze. J Allergy Clin Immunol. (2018) 143:1607–10. 10.1016/j.jaci.2018.12.985 30579850

[45] Zinelli C Caffarelli C Strid J Jaffe A Atherton DJ. Measurement of nitric oxide and 8-isoprostane in exhaled breath of children with atopic eczema. Clin Exp Dermatol. (2009) 34:607–12. 10.1111/j.1365-2230.2008.03142.x 19508477

[46] Mastrorilli C Posa D Cipriani F Caffarelli C. Asthma and allergic rhinitis in childhood: what's new. Pediatr Allergy Immunol. (2016) 27:795–803. 10.1111/pai.12681 27862336

[47] Caffarelli C Garrubba M Greco C Mastrorilli C Povesi Dascola C Asthma and food allergy in children: is there a connection or interaction? Front Pediatr. (2016) 4:34 10.3389/fped.2016.00034 27092299 PMC4821099

[48] Caffarelli C Dondi A Povesi Dascola C Ricci G. Skin prick test to foods in childhood atopic eczema: pros and cons. Ital J Pediatr. (2013) 39:48. 10.1186/1824-7288-39-48 23902622 PMC3734168

[49] Du X Wang L Wu S Yuan L Tang S Xiang Y. Efficacy of probiotic supplementary therapy for asthma, allergic rhinitis, and wheeze: a meta-analysis of randomized controlled trials. Allergy Asthma Proc. (2019) 40:250–60. 10.2500/aap.2019.40.4227 31262380

[50] Del-Rio-Navarro BE Espinosa-Rosales FJ Flenady V Sienra-Monge JJL Immunostimulants for preventing respiratory tract infection in children. Evid Based Child Health. (2012) 7:629–717. 10.1002/ebch.1833 PMC13178731 17054227

[51] Caffarelli C Cardinale F Povesi-Dascola C Dodi I Mastrorilli V Ricci G. Use of probiotics in pediatric infectious diseases. Expert Rev Anti Infect Ther. (2015) 13:1517–35. 10.1586/14787210.2015.1096775 26496433

[52] Ducharme FM Tse SM Chauhan B. Diagnosis, management, and prognosis of preschool wheeze. Lancet. (2014) 383:1593–604. 10.1016/S0140-6736(14)60615-2 24792856

[53] Kaiser SV Huynh T Bacharier LB Rosenthal JL Bakel LA Parkin PC. Preventing exacerbations in preschoolers with recurrent wheeze: a meta-analysis. Pediatrics. (2016) 137:2015–4496. 10.1542/peds.2015-4496 27230765

[54] Fitzpatrick AM Jackson DJ Mauger DT Boehmer SJ Phipatanakul W Sheehan WJ. Individualized therapy for persistent asthma in young children. J Allergy Clin Immunol. (2016) 138:1608–18. 10.1016/j.jaci.2016.09.028 27777180 PMC5148729

[55] British Thoracic Society Scottish Intercollegiate Guidelines Network British Guideline on the Management of Asthma: A National Clinical Guideline. (SIGN 158) (2019). Available online at: https://www.sign.ac.uk/sign-158-british-guideline-on-the-management-of-asthma (accessed April 10, 2020).

[56] National Institute for Health and Care Excellence Asthma: Diagnosis, Monitoring and Chronic Asthma Management. (2020). Available online at: www.nice.org.uk/guidance/ng 80 (accessed April 10, 2020).

[57] Castro-Rodriguez JA Rodrigo GJ. Efficacy of inhaled corticosteroids in infants and preschoolers with recurrent wheezing and asthma: a systematic review with meta-analysis. Pediatrics. (2009) 123:519–25. 10.1542/peds.2008-2867 19254986

[58] Jochmann A Artusio L Robson K Nagakumar P Collins N Fleming L. Infection and inflammation in induced sputum from preschool children with chronic airways diseases. Pediatr Pulmonol. (2016) 51:778–86. 10.1002/ppul.23366 26678320

[59] Global Initiative for Asthma Global Strategy for Asthma Management and Prevention. (2019). Available online at: www.gina.org (accessed March 13, 2020).

[60] Brand PL Caudri D Eber E Gaillard EA Garcia-Marcos L Hedlin G. Classification and pharmacological treatment of preschool wheezing: changes since 2008. Eur Respir J. (2014) 43:1172–7. 10.1183/09031936.00199913 24525447

[61] Bisgaard H Gillies J Groenewald M Maden C. The effect of inhaled fluticasone propionate in the treatment of young asthmatic children. A dose comparison study. Am J Respir Crit Care Med. (1999) 160:126–31. 10.1164/ajrccm.160.1.9811024 10390389

[62] Klok T Kaptein AA Duiverman EJ Brand PL. It's the adherence, stupid (that determines asthma control in preschool children)! Eur Respir J. (2014) 43:783–91. 10.1183/09031936.00054613 23845718

[63] Castro-Rodriguez JA Rodrigo GJ. b-agonists through metered-dose inhaler with valved holding chamber versus nebulizer for acute exacerbation of wheezing or asthma in children under 5 years of age: a systematic review with meta-analysis. J Pediatr. (2004) 145:172–7. 10.1016/j.jpeds.2004.04.007 15289762

[64] Food and Drug Administration FDA Requires Boxed Warning About Serious Mental Health Side Effects for Asthma and Allergy Drug Montelukast (Singulair); Advises Restricting Use for Allergic Rhinitis. (2020). Available online at: https://www.fda.gov/drugs/drug-safety-and-availability/fda-requires-boxed-warning-about-serious-mental-health-side-effects-asthma-and-allergy-drug (accessed April 10, 20202).

[65] Pajno GB Bernardini R Peroni D Arasi S Martelli A Landi M. Clinical practice recommendations for allergen-specific immunotherapy in children: the Italian consensus report. Ital J Pediatr. (2017) 43:13. 10.1186/s13052-016-0315-y 28257631 PMC5347813

[66] Di Rienzo V Cadario G Grieco T Galluccio AG Caffarelli C Liotta G. Sublingual immunotherapy in mite-sensitized children with atopic dermatitis: a randomized, open, parallel-group study. Ann Allergy Asthma Immunol. (2014) 113:671–3. 10.1016/j.anai.2014.09.009 25304342

[67] Franceschini F Bottau P Caimmi S Cardinale F Crisafulli G Liotti L. Evaluating children with suspected allergic reactions to vaccines for infectious diseases. Allergy Asthma Proc. (2018) 39:177–83. 10.2500/aap.2018.39.4128 29669664

[68] Castro-Rodriguez JA Abarca K Forno E. Asthma and the risk of invasive pneumococcal disease: a meta-analysis. Pediatrics. (2020) 145:e20191200. 10.1542/peds.2019-1200 31843863 PMC6939845

[69] Vrijlandt E El Azzi G Vandewalker M Rupp N Harper T Graham L. Safety and efficacy of tiotropium in children aged 1–5 years with persistent asthmatic symptoms: a randomised, double-blind, placebo-controlled trial. Lancet Respir Med. (2018) 6:127–37. 10.1016/S2213-2600(18)30012-2 29361462

[70] Zeiger RS Mauger D Bacharier LB Guilbert TW Martinez FD Lemanske RF Jr. Daily or intermittent budesonide in preschool children with recurrent wheezing. N Engl J Med. (2011) 365:1990–2001. 10.1056/NEJMoa1104647 22111718 PMC3247621

[71] McKean M Ducharme F Inhaled steroids for episodic viral wheeze of childhood. Cochrane Database Syst Rev. (2000) 2:CD001107 10.1002/14651858.CD001107 PMC8406470 10796596

[72] Chong J Haran C Chauhan BF Asher I Intermittent inhaled corticosteroid therapy versus placebo for persistent asthma in children and adults. Cochrane Database Syst Rev. (2015) 7:CD011032 10.1002/14651858.CD011032.pub2 PMC8676065 26197430

[73] Papi A Nicolini G Baraldi E Boner AL Cutrera R Rossi GA. Regular vs prn nebulized treatment in wheeze preschool children. Allergy. (2009) 64:1463–71. 10.1111/j.1398-9995.2009.02134.x 19772514

[74] Chauhan BF Chartrand C Ducharme FM Intermittent versus daily inhaled corticosteroids for persistent asthma in children and adults. Cochrane Database Syst Rev. (2013) 2:CD009611 10.1002/14651858.CD009611.pub3 PMC11627141 23450606

[75] Rodrigo GJ Castro-Rodriguez JA. Daily vs. intermittent inhaled corticosteroids for recurrent wheezing and mild persistent asthma: a sistematic review with meta-analysis. Respir Med. (2013) 107:1133–40. 10.1016/j.rmed.2013.05.005 23769720

[76] de Benedictis FM Bush A. Infantile wheeze: rethinking dogma. Arch Dis Child. (2017) 102:371–5. 10.1136/archdischild-2016-311639 27707694

[77] Castro-Rodriguez JA Custovic A Ducharme FM. Treatment of asthma in young children: evidence-based recommendations. Asthma Res Pract. (2016) 2:5. 10.1186/s40733-016-0020-z 27965773 PMC5142379

[78] Clavenna A Sequi M Cartabia M Fortinguerra F Borghi M Bonati M. Effectiveness of nebulized beclomethasone in preventing viral wheezing: an RCT. Pediatrics. (2014) 133:505–12. 10.1542/peds.2013-2404 24534400

[79] Knorr B Franchi LM Bisgaard H Vermeulen JH LeSouef P Santanello N. Montelukast, a leukotriene receptor antagonist, for the treatment of persistent asthma in children aged 2 to 5 years. Pediatrics. (2001) 108:E48. 10.1542/peds.108.3.e48 11533366

[80] Brodlie M Gupta A Rodriguez-Martinez CE Castro-Rodriguez JA Ducharme FM McKean MC Leukotriene receptor antagonists as maintenance and intermittent therapy for episodic viral wheeze in children. Cochrane Database Syst Rev. (2015) 10:CD008202 10.1002/14651858.CD008202.pub2 PMC6986470 26482324

[81] Krawiec M Strzelak A Krenke K Modelska-Wozniak I Jaworska J Kulus M Fluticasone or Montelukast in preschool wheeze. Clin Pediatr. (2015) 54:273–81. 10.1177/0009922814550158 25246602

[82] Szefler SJ Baker JW Uryniak T Goldman M Silkoff PE. Comparative study of budesonide inhalation suspension and montelukast in young children with mild persistent asthma. J Allergy Clin Immunol. (2007) 120:1043–50. 10.1016/j.jaci.2007.08.063 17983871

[83] Nwokoro C Pandya H Turner S Eldridge S Griffiths CJ Vulliamy T. Intermittent montelukast in children aged 10 months to 5 years with wheeze (WAIT trial): a multicentre, randomised, placebo-controlled trial. Lancet Respir Med. (2014) 2:796–803. 10.1016/S2213-2600(14)70186-9 25212745 PMC4189104

[84] Grigg J Nibber A Paton JY Chisholm A Guilbert TW Kaplan A. Matched cohort study of therapeutic strategies to prevent preschool wheezing/asthma attacks. J Asthma Allergy. (2018) 11:309–21. 10.2147/JAA.S178531 30588038 PMC6294169

[85] Bloom CI Saglani S Feary J Jarvis D Quint JK. Changing prevalence of current asthma and inhaled corticosteroid treatment in the UK: population-based cohort 2006-2016. Eur Respir J. (2019) 53:1802130. 10.1183/13993003.02130-2018 30765507

[86] Mikalsen IB Dalen I Karlstand O Eide GE Magnus M Nystad W. Airwy symptoms and atopy in young children prescribed asthma medications: a large-scale cohort study. Pediatr Pulmonol. (2019) 54:1557–66. 10.1002/ppul.24437 31273956

[87] Nardini G Borrelli M Santamaria F Asthma treatment of pediatric airway disorders: Choose wisely! Pediatr Pulmonol. (2020) 55:11–3. 10.1002/ppul.24550 31710174

[88] Guilbert TW Morgan WJ Zeiger RS Mauger DT Boehmer SJ Szefler SJ. Long-term inhaled corticosteroids in preschool children at high risk for asthma. N Engl J Med. (2006) 354:1985–97. 10.1056/NEJMoa051378 16687711

[89] Murray CS Woodcock A Langley SJ Morris J Custovic A Secondary prevention of asthma by the use of inhaled fluticasone dipropionate in wheezy infants (IFWIN): double-blind, randomised controlled study. Lancet. (2006) 368:754–62. 10.1016/S0140-6736(06)69285-4 16935686

[90] Bisgaard H Hermansen MN Loland L Halkjaer LB Buchvald F. Intermittent inhaled corticosteroids in infants with episodic wheezing. N Engl J Med. (2006) 354:1998–2005. 10.1056/NEJMoa054692 16687712

[91] Fuhlbrigge AL Kelly HW. Inhaled corticosteroids in children: effects on bone mineral density and growth. Lancet Respir Med. (2014) 2:487–96. 10.1016/S2213-2600(14)70024-4 24717638

[92] Kelly HW Sternberg AL Lescher R Fuhlbrigge AL Williams P Zeiger RS. Effect of inhaled glucocorticoids in childhood on adult height. N Engl J Med. (2012) 367:904–12. 10.1056/NEJMoa1203229 22938716 PMC3517799

[93] Zhang L Belizario Lasmar L Castro-Rodriguez JA The impact of asthma and its treatment on growth: an evidence-based review. J Pediatr. (2019) 1:10–22. 10.1016/j.jped.2018.10.005 30472355

[94] Salvatoni A Piantanida E Nosetti L Nespoli L. Inhaled corticosteroids in childhood asthma: long-term effects on growth and adrenocortical function. Paediatr Drugs. (2003) 5:351–61. 10.2165/00128072-200305060-00001 12765485

[95] Patel L Wales JK Kibirige MS Massarano AA Couriel JM Clayton PE. Symptomatic adrenal insufficiency during inhaled corticosteroid treatment. Arch Dis Child. (2001) 85:330–4. 10.1136/adc.85.4.330 11567945 PMC1718935

[96] Saglani S Malmström K Pelkonen AS Malmberg LP Lindahl H Kajosaari M. Airway remodeling and inflammation in symptomatic infants with reversible airflow obstruction. Am J Respir Crit Care Med. (2005) 171:722–7. 10.1164/rccm.200410-1404OC 15657459

